# Spanish-language text classification for environmental evidence synthesis using multilingual pre-trained models

**DOI:** 10.1186/s13750-025-00370-9

**Published:** 2025-11-12

**Authors:** Violeta Berdejo-Espinola, Ákos Hajas, Richard Cornford, Nan Ye, Tatsuya Amano

**Affiliations:** 1 School of the Environment, The University of Queensland, Brisbane, Australia; 2 Centre for Biodiversity and Conservation Science, The University of Queensland, Brisbane, Australia; 3 Independent Researcher, Brisbane, Australia; 4 International Institute for Applied Systems Analysis, Laxenburg, Austria; 5 School of Mathematics and Physics, The University of Queensland, Brisbane, Australia

**Keywords:** Natural language processing, Non-English, Evidence synthesis, Biodiversity conservation, Language barriers, Explainable AI, SHAP, Multilingual language model

## Abstract

Artificial intelligence (AI) is increasingly being explored as a tool to optimize and accelerate various stages of evidence synthesis. A persistent challenge in environmental evidence syntheses is that these remain predominantly monolingual (English), leading to biased results and misinforming cross-scale policy decisions. AI offers a promising opportunity to incorporate non-English language evidence in evidence syntheses screening process and help to move beyond the current monolingual focus of evidence syntheses. Using a corpus of Spanish-language peer-reviewed papers on biodiversity conservation interventions, we developed and evaluated text classifiers using supervised machine learning models. Our best-performing model achieved 100% recall meaning no relevant papers (*n* = 9) were missed and filtered out over 70% (*n* = 867) of negative documents based only on the title and abstract of each paper. The text was encoded using a pre-trained multilingual model and class-weights were used to deal with a highly imbalanced dataset (0.79%). This research therefore offers an approach to reducing the manual, time-intensive effort required for document screening in evidence syntheses—with minimal risk of missing relevant studies. It highlights the potential of multilingual large language models and class-weights to train a light-weight non-English language classifier that can effectively filter irrelevant texts, using only a small non-English language labelled corpus. Future work could build on our approach to develop a multilingual classifier that enables the inclusion of any non-English scientific literature in evidence syntheses.

**Supplementary Information:**

The online version contains supplementary material available at 10.1186/s13750-025-00370-9.

## Resumen

La inteligencia artificial (IA) se está explorando cada vez más como una herramienta para optimizar y acelerar diversas etapas de la síntesis de evidencia. Un desafío persistente en la síntesis de evidencia ambiental es que estas son predominantemente monolingües (inglés), lo que conduce a resultados sesgados y a decisiones políticas erróneas. La IA ofrece una oportunidad prometedora para incorporar evidencia científica en idiomas distintos del inglés en el primer paso del proceso de la síntesis de evidencia, la selección de documentos relevantes, contribuyendo a ir más allá del enfoque monolingüe actual de la síntesis de evidencia. Utilizando un corpus de artículos revisados por pares en español sobre intervenciones de conservación de la biodiversidad, desarrollamos y evaluamos clasificadores de texto utilizando modelos de aprendizaje automático supervisado (en inglés, “supervised machine learning”). Nuestro mejor modelo alcanzó un 100% de exhaustividad (en inglés, “recall”), lo que significa que no se pasó por alto ningún artículo relevante (*n* = 9) y se filtraron más del 70% (*n* = 867) de los documentos negativos basándose únicamente en el título y el resumen de cada artículo. El texto se codificó utilizando un modelo multilingüe pre-entrenado y se utilizaron ponderaciones de clase para tratar un conjunto de datos muy desequilibrado (0.79%). Nuestro trabajo destaca el potencial de los modelos lingüísticos multilingües pre-entrenados y los pesos de clase para entrenar un clasificador ligero de idiomas distintos del inglés con la capacidad de filtrar eficazmente los textos irrelevantes, utilizando solo un pequeño corpus de documentos etiquetado. Futuras investigaciones podrían partir de nuestro enfoque para desarrollar un clasificador multilingüe que permita incluir cualquier literatura científica en idiomas distintos del inglés en las síntesis de pruebas.

## 要約 – 日本語

人工知能（AI）は、エビデンスの統合における様々な段階を最適化・加速するツールとして、ますます活用が進められている。環境分野では、エビデンス統合が主に英語のみで行われ、結果に偏りが生じることで政策決定に対して誤った情報を伝え得ることが、継続した課題となっている。AIは、非英語の文献をエビデンス統合のスクリーニング過程に取り入れるために有望な手段であり、現在の英語偏重のエビデンス統合を大きく改善する可能性を秘めている。本研究では、生物多様性の保全活動に関するスペイン語の査読付き論文コーパスを用いて、教師あり機械学習モデルによるテキスト分類の開発・評価を行った。最も性能の高かったモデルは、タイトルと要旨の情報のみを利用することで再現率（recall）100%を達成し、関連した文献（n=9）を一つも見逃すことなく、関連していない文献の70%以上（n=867）を除外することができた。このモデルでは、テキストは事前学習済みの多言語モデルを用いてエンコードされ、極端に不均衡なデータセット（0.79%）に対応するためにクラス重み付けが用いられた。本研究は、エビデンス統合において手作業で行なう文献スクリーニングが必要とする時間と労力を、関連研究を見逃すリスクを最小限に抑えつつ軽減するアプローチを提供する。また、多言語の大規模言語モデルとクラス重み付けの活用により、少量の非英語ラベル付きコーパスのみで、不要なテキストを効果的に除外できる軽量な非英語テキスト分類を実現する可能性を示している。今後の研究では、本アプローチを発展させ、あらゆる非英語の科学文献をエビデンス統合に取り込むことが可能な多言語テキスト分類の開発が期待される。

## Background

Synthesising scientific evidence in an unbiased and comprehensive way—for example through systematic reviews and mapping—is fundamental to inform evidence-based conservation and thus devise solutions to the current biodiversity crisis. Incorporating multilingual evidence is crucial for evidence-based conservation, as systematically excluding non-English literature limits comprehensiveness and reduces the ability of syntheses to account for biases. Incomplete evidence syntheses lead to flawed decisions and policies [1, 2] and misinform environmental governance at both local and global scales [3].

To date, evidence syntheses in environmental sciences have remained predominantly monolingual (English) [2, 4]. For example, over 60% of the systematic reviews and maps published in *Environmental Evidence* exclusively searched for English-language evidence. Similarly, only 4% of the evidence used in global assessments by the Intergovernmental Platform on Biodiversity and Ecosystem Services (IPBES) were in non-English language [2, 4, 5]. This monolingual approach could have multiple consequences for evidence synthesis. First, English-only evidence synthesis excludes the substantial body of scientific evidence published in non-English languages [6, 7]. For instance, non-English-language literature captures a greater amount of data sources than English-language evidence on the economic cost caused by invasive species worldwide [8]. Second, by ignoring non-English-language evidence we could overlook locally specific and context-relevant evidence [6], which is typically preferred by conservation policy-makers [8, 9]. On average, non-English-language literature constitutes 65% of the references cited in national biodiversity conservation assessments, and these are recognized as relevant knowledge sources by 75% of report authors in countries where English is not an official language [9]. Finally, ignoring non-English-language studies can lead to systematic biases in statistical results, as statistically more significant and positive results are more likely to be published in English [10, 11]. Together, these consequences could undermine the quality of meta-analyses, scientific conclusions, and policy recommendations, particularly in regions where local knowledge and context-specific research published in native languages provide crucial insights that are not captured in the international English-language literature.

The time-consuming and labour-intensive nature of evidence synthesis, often poses a challenge in including non-English-language evidence. For example, manually completing a systematic map in environmental sciences is estimated to take 211 days full-time equivalent for an experienced reviewer, with roughly 91 days dedicated only to screening stages [12]. The amount of time and people required to conduct evidence synthesis can be much larger if multiple languages are considered in the synthesis. Indeed a survey with authors of 72 systematic reviews and maps published in *Environmental Evidence* showed that the lack of time, relevant language skills, and necessary resources is the main reason for them not to include non-English-language evidence in their studies [5]. Furthermore, a synthesis on the effectiveness of biodiversity conservation interventions conducted in 17 languages required the collaboration of 38 people and the involvement of two institutions for over two years to cover scientific data of journals from 28 countries [6], highlighting the large efforts needed to make evidence synthesis multilingual.

Thanks to recent developments in artificial intelligence (AI), researchers have increasingly been exploring their integration in various stages of evidence synthesis [13–17]. Traditionally, classification-based approach using machine learning classifiers like logistic regression, naïve bayes, support vector machines, and more recently neural networks have been applied to automatically identify evidence that is relevant to a set of eligibility criteria in the ecological and health domain [14–16], with some automated classifiers performing better than manual screening [15]. For instance, a classification pipeline including machine learning and active learning can find 95% of eligible studies after screening between only 8–33% of the studies [15]. Further, the same pipeline can find from 70 to 100% of relevant studies after screening only 10% of the abstracts [15]. With the recent advances of generative AI, researchers can do end to evidence synthesis achieving varying levels of accuracy in the different stages of the evidence synthesis process [17–20]. Virtual AI assistants (but not using Large language models (LLM) reasoning capabilities) have been found to help human reviewers with search string development and the screening of article titles and abstracts [17]. LLMs like Claude, ChatGPT, and the Bing AI Chat tool can act as second reviewers and are able to extract and tabulate valuable information from scientific articles (including PDFs), e.g. geographic location, taxonomic information and other study characteristics [21–24]. Careful use of LLMs for evidence synthesis is however required, as outputs can be incomplete and biased, or even contain ‘hallucinations’ (fabricated data) [18, 21]. Nonetheless, recent progress in generative AI, including complex reasoning capabilities and the ability to retrieve information from the internet [25] highlight the huge potential of AI to accelerate evidence synthesis workflows.

Despite the promise of machine learning and natural language processing algorithms, most current proposed solutions for automatically identifying relevant literature are trained on English-language text, limiting the potential for (semi-)automated multilingual evidence synthesis. Yet, pre-trained multilingual language models (e.g. mBERT, XLM-R, and mT5 [26–28]) are increasingly available, covering over 100 languages and displaying high accuracy when fine-tuned on downstream tasks, such as classification, summarisation and question answering. Thus, developing text classifiers trained on non-English language scientific literature has potential to both widen information coverage and reduce screening times for multilingual evidence syntheses, allowing for improved use of non-English-language evidence.

Using a multilingual global database of scientific peer-reviewed articles on the effectiveness of biodiversity conservation interventions identified based on a set of selection criteria (i.e., inclusion/exclusion –see selection criteria in ( [6, 29]), this study develops supervised machine learning to classify Spanish-language literature that is relevant to the same selection criteria. We aim to (i) determine the best performing models for classifying relevant Spanish-language literature and (ii) identify the aspects of feature engineering and feature extraction that influence the performance of classification models. The importance of Spanish-language studies for conservation is unquestionable; up to 13% of the scientific literature on conservation is in Spanish [30], and over 6% of the global population are Spanish native speakers with most of these people living in Latin America [31], a region that houses seven biodiversity hotspots (i.e., Atlantic Forest, the Caribbean, the Cerrado, Mesoamerica, the Valdivian temperate rainforest, the Tropical Andes, and Tumbes-Chocó-Magdalena). Thus, exploring ways to access the knowledge produced in the Spanish language is fundamental to foster inclusive, effective, and locally informed evidence-based conservation. We also anticipate that the approach developed in this study will be readily transferable to other non-English languages.

Box 1. Glossary

- Supervised machine learning: modelling approach that uses human-labelled input data to learn the underlying relationships between inputs and outputs. The trained model is able to predict correct outputs based on new, unlabelled data.
- Embeddings: numerical representations of text data that capture semantic relationships.
- Pre-trained language models: neural networks trained on massive text datasets, enabling them to understand human language.
- Hyperparameter tuning: the process of finding the optimal set of hyperparameters for a machine learning model before training.
- Ablation studies: consists of systematically removing components of a model to assess their individual contributions to overall performance to understand which parts of the model are essential and which might be redundant.

## Methodology

We compared three supervised binary classifiers: logistic regression (LR), support vector machine (SVM), and multi-layer perceptron (MLP). We used different combinations of classifiers, feature extraction, and data balancing approaches to assess how these factors impact the performance of the classification models. Each document’s text length includes the title and abstract of a scientific article. In total, 38 model variants were generated (Table S4).

## Pre-processing training data

**Fig. 1:**
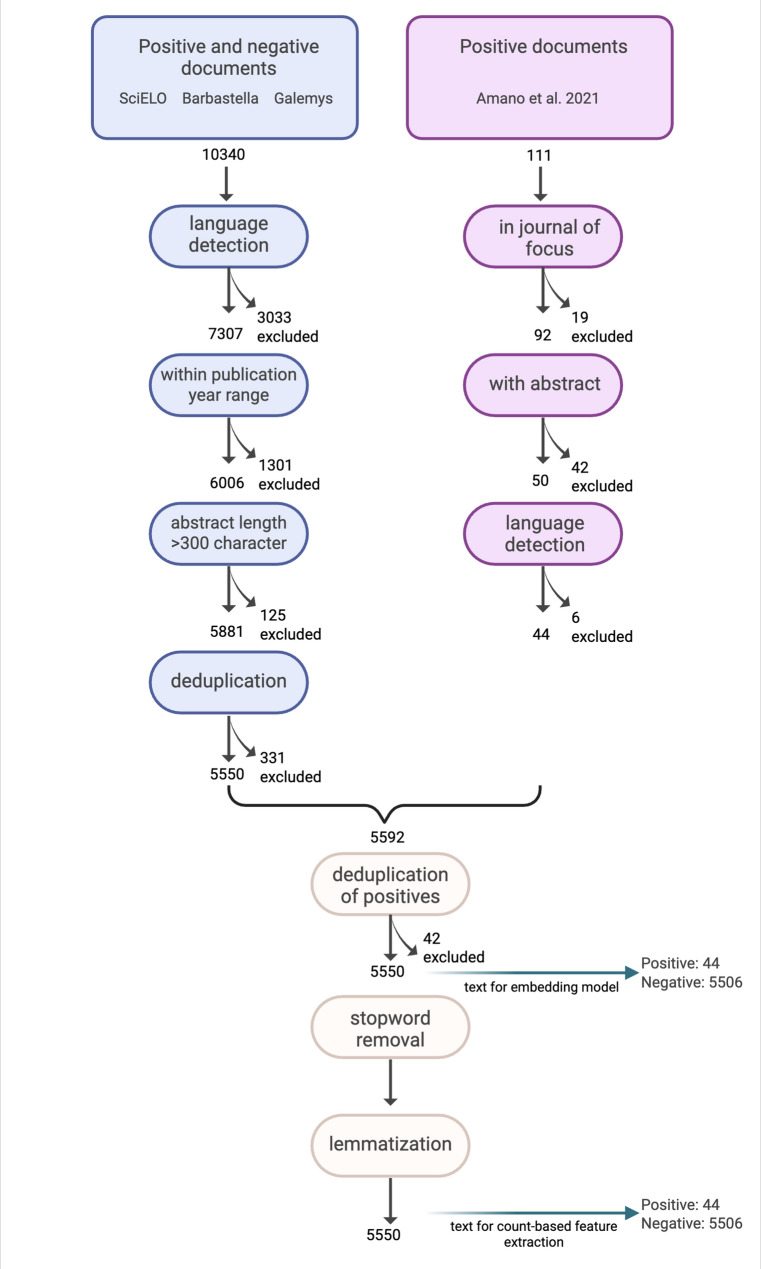
Flowchart showing the retrieval and pre-processing of training data and the number of studies included and excluded at each stage of the pre-processing. Green arrows output the final number of documents in the positive and negative class. Journals of focus are *Acta Zoológica Mexicana*,* Barbastella*,* Ecología Aplicada*,* Ecología Austral*,* Galemys*,* Huitzil*,* Madera y Bosques*,* Mastozoología Neotropical*,* Quebracho*,* Revista Chilena de Historia Natural*,* Revista de Biología Tropical*,* Revista Mexicana de Biodiversidad*,* Revista Mexicana de Ciencias Forestales*, and *Therya.* Created in https://BioRender.com

We define relevant documents (i.e. articles) as studies that tested the effectiveness of a conservation actions on biodiversity outcomes and were published in Spanish (i.e., the title, abstract, and main text is written in Spanish). These documents were identified through a discipline-wide multilingual synthesis [6], which screened 26,819 documents published in 56 Spanish-language journals across 11 countries including Argentina, Chile, Colombia, Costa Rica, Dominican Republic, Ecuador, El Salvador, Mexico, Nicaragua, Peru, Uruguay for specific year ranges until 2019 (see year ranges and more information in Sup. Mat. Table 1). Amano et al. (2021) [6] identified 111 relevant Spanish-language documents (Fig. 1) covering conservation actions, such as species re-introduction programs, ecological restoration, reforestation, control of invasive species, installation of bat and bird nest-boxes, fire management programs, agricultural land use programs for forest conservation, community forest management programs, and more.

We restricted the scope of this study to documents from 12 journals indexed in SciELO (https://scielo.org/es/*)*, a regional language-specific repository, in which documents are open access and largely available on the website, as well as two other journals that had a high number of relevant documents in [6]. As a result, documents from 14 journals within the year ranges screened in [6] were included in our analysis (*Acta Zoológica Mexicana*,* Barbastella*,* Ecología Aplicada*,* Ecología Austral*,* Galemys*,* Huitzil*,* Madera y Bosques*,* Mastozoología Neotropical*,* Quebracho*,* Revista Chilena de Historia Natural*,* Revista de Biología Tropical*,* Revista Mexicana de Biodiversidad*,* Revista Mexicana de Ciencias Forestales*,* Therya)* (Fig. 1). Custom scrapers were written in Python language (https://github.com/hakosh/journal-scraper) to retrieve all documents published in the 14 journals by selecting those that met the following selection criteria: (i) title and abstract should be available in Spanish, but there could also be an English or Portuguese version, (ii) title and abstract should be available on the website not in PDF format, and (iii) the main text should be in Spanish only. A total of 10,340 documents were retrieved in HTML format, cleaned up, and relevant information including the title and abstract were extracted. Next, the text was processed using a language detection model—fastText [32]—and 3,033 documents were removed as they had the main text in English in addition to Spanish (Fig. 1). We excluded 1,301 documents that were outside the year range screened in [6]. Additionally, 125 documents were removed as their abstract was shorter than 300 characters. Using Polars String methods [33], we removed 331 documents that were duplicates or editorials, erratum, *In Memoriam*, or retracted documents, leaving 5,550 documents. Out of the 111 relevant Spanish-language documents found in [6], we excluded 19 documents as they were not published in the journals of focus, 42 documents didn’t have an abstract, and six documents also had their main text available in English. As a result, we used 44 relevant documents as positive documents in our study.

Articles in the scraped dataset (from SciELO, *Barbastella*, and *Galemys*) that were present in our collection of positive documents were removed. We identified these duplicates using article titles and the Levenshtein distance, a string metric that measures the minimum number of single-character edits (insertions, deletions, or substitutions) required to change one word into the other. We set the similarity score above 0.95 using the Levenshtein Python module [34] as this was the threshold in which all the positive titles in the body of documents scraped from SciELO, *Barbastella*, and *Galemys*, were ‘similar enough’ to the titles in the corpus of positive documents. The remaining documents published in the subset of focus journals were considered negative (i.e., non-relevant) documents and annotated accordingly with positive or negative labels. Our final corpus consisted of 5,550 documents, with 44 positive documents and 5,506 negative documents.

Finally, the corpus was pre-processed for two feature extraction approaches (i.e., term frequency and term frequency inverse-document frequency). We removed numbers, special characters, punctuations, stop-words i.e., words that hold little information (e.g., ‘el’, ‘la’, ‘y’, ‘en’ in Spanish), and extra white spaces (Fig. 1). We used the Python stop_word module as it had the most comprehensive predefined list of stop words. We lemmatized words to remove inflectional endings (e.g., -o, -a, -s, -es in Spanish) and to return the base or dictionary form of a word, known as the *lemma* [35], using a Spanish language pre-trained model (‘es_core_news_md’) from the spaCy library [36].

## Feature extraction

We used three approaches to extract features from the text in documents: (i) term frequency (TF), (ii) term frequency inverse document frequency (TF-IDF) and (iii) sentence-level embeddings. Term frequency is the number of times a word occurs in a document. TF-IDF weights term frequency by the number of documents that term occurs in, down-weighting common terms [37]. These term-based features represent text without accounting for word position or semantic meaning, i.e., ‘bag of words’. In contrast, our third approach is context-aware and derives semantically meaningful sentence embeddings using the SentenceTransformers encoder (a.k.a SBERT) [38, 39] implemented through Sentence Encoder (https://github.com/koaning/embetter). The sentence embeddings learned from a large multilingual corpora, which consist of text data from various languages. During training, the model learns to predict the context of words or phrases in multiple languages, effectively capturing the semantic relationships between words across languages. We tested the performance of two multilingual pre-trained models when mapping sentences and paragraphs into vectors (*distiluse-base-multilingual-cased-v1* and *paraphrase-multilingual-mpnet-base-v2*) Table 1.

**Table 1:** Implemented classifiers, feature extraction techniques, and balance strategies

Classifier	Feature extraction	Balancing approach
Logistic regression Support vector machine Multi-layer perceptron	TF (term frequency) TF-IDF (term frequency inverse document frequency) Sentence embedding	No sampling Random undersampling Random upsampling Synthetic upsampling Class weights

Note that not all combinations are possible. For example, the multi-layer perceptron classifier cannot handle class weights. See table S4 for all 38 models used in this study

All text pre-processing, model training, and testing was conducted in Python 3.11 [40] using modules including NumPy [41], Polars [33], Pandas [42], matplotlib [43], NLTK [44], Levenshtein [34], spaCy [36], scikit-learn [45], imbalanced-learn [46], embetter (https://github.com/koaning/embetter*)*, SentenceTransformer [38], PyTorch [47].

## Accounting for imbalanced data

Because positive documents only account for 0.79% of the data, we tested four approaches to handle our imbalanced dataset: weighting the model loss function, random oversampling of documents of the minority class (positives/relevant), synthetically oversampling documents of the minority class using the ADASYN algorithm, and random undersampling of documents of the majority class (negative/irrelevant). In a weighted loss function, the weights are used to make the model more sensitive to the minority class by increasing the cost of a misclassification of that class (see formulas for weight calculations in Sup. Mat.). On the other hand, resampling can add samples from the minority class or remove samples from the majority class in an effort to balance the classes. RandomUnderSampler, RandomOverSampler, and ADASYN of the imblearn module within the scikit-learn library were used to resample the training data and were all randomly seeded at 42.

## Classifiers and hyperparameters

We fitted multiple classification heads of three model families: logistic regression, support vector machine (SVM), and multilayer perceptron to evaluate their performance when classifying text. We trained ‘baseline’ models using their default hyperparameters except for the multilayer perceptron; we changed the activation function to ‘logistic’ (instead of the default ‘relu’), and it had no hidden layers. To ensure reproducibility, we seeded all random initialisations at 42. We used LogisticRegression, SVC, and MLPClassifier within the scikit-learn library.

## Training and testing

In all model families, training, development, and testing sets were created to test the validity of the classifier. We split the corpus stratifying classes resulting in 80% of the corpus as training data (*n* = 4,440) and the remaining 20% was retained for testing the model (*n* = 1,110). To assess model performance during training, we used stratified two-fold cross-validation and to enable direct comparisons of model performance, all models were cross-validated using the same data subsets. To avoid over-fitting, development sets were used to evaluate classification performance, whilst the testing sets were used to evaluate the classifier’s performance when applied to unseen data. We investigated the performance of the best performing model by training the model on four different random splits and calculated the standard error of the training set losses (Sup. Mat. Table 3). To make predictions in a systematic and reproducible way, we used scikit-learn [45] pipelines to transform and resample the data and fit estimators. We used the cross_val_predict and StratifiedKFold functions within the scikit-learn [45] library for stratified cross-validation and the Pipeline and make_pipeline functions from the imbalanced-learn [46] library for implementing pipelines consisting of data transformations and a final classifier.

## Evaluation

The models were evaluated using the precision (i.e., proportion of all the model’s positive classifications that are actually positive), recall (i.e., proportion of all actual positives that were classified correctly as positives), and F1 scores (i.e., the harmonic mean of precision and recall). Besides these metrics, we generated confusion matrices. Because the classifiers developed here were primarily designed to avoid missing any potentially eligible study, we evaluated the model with the highest cross-validation F1 among those attaining a threshold test set recall value above 90%. We conducted ablation studies by systematically removing or replacing modules (i.e., encoding and weighting approach) from the model architecture to assess their individual contributions to the overall performance.

## Hyperparameter tuning

We searched for the best set of hyperparameters in the Logistic Regression and SVM to optimize model performance. We tested different solvers, including ‘liblinear’ and ‘lbfgs’. We also tested different values of the regularization parameter C that controls the strength of Ridge Regression or Tikhonov regularization applied to the model. Smaller C values add penalties to large weights.

## Explainability and error analysis

We explained predictions using SHapley Additive Explanations (SHAP) [48], a feature-based (i.e., word-based) interpretability method that can be integrated into supervised classification tasks. SHAP is based on the Shapley Values used in game theory. The approach measures the relative contribution of each feature (i.e., word or token) to the output produced by the classification model by assigning a value to each feature in a specific prediction. Each prediction (i.e., f(x)) is calculated as:

$$\:f\left(inputs\right)=basevalue+\sum\:\left(SHAP\:values\:of\:\:features\right)$$

The base value (expected value) is the model’s prior belief and represents the average prediction (expressed in SVM decision scores) the model would make for any given text if it didn’t have any specific information from the current text. SHAP calculations start from the base value. Next, each feature in the text is assigned a SHAP value, and the sum of these features’ SHAP values (*f(inputs)*) are the contributions that adjust the prediction higher or lower relative to the baseline. The sum of the SHAP values for all features, when added to the base value, equals the final SVM decision score prediction for the given text. It is important to highlight that these explanations come with some limitations. For instance, the observed SHAP values are approximations as the exact calculation of Shapley values is computationally infeasible due to the exponential number of feature combinations that need to be evaluated [48].

Additional qualitative evaluation was conducted by inspecting whether the words assigned a high relevance by the model were associated with the impact class. For this, we created word clouds of all predictions to gain insights from the most frequently used words (Sup. Mat. Figure 1).

## Results

### Classifier performance

**Fig. 2:**
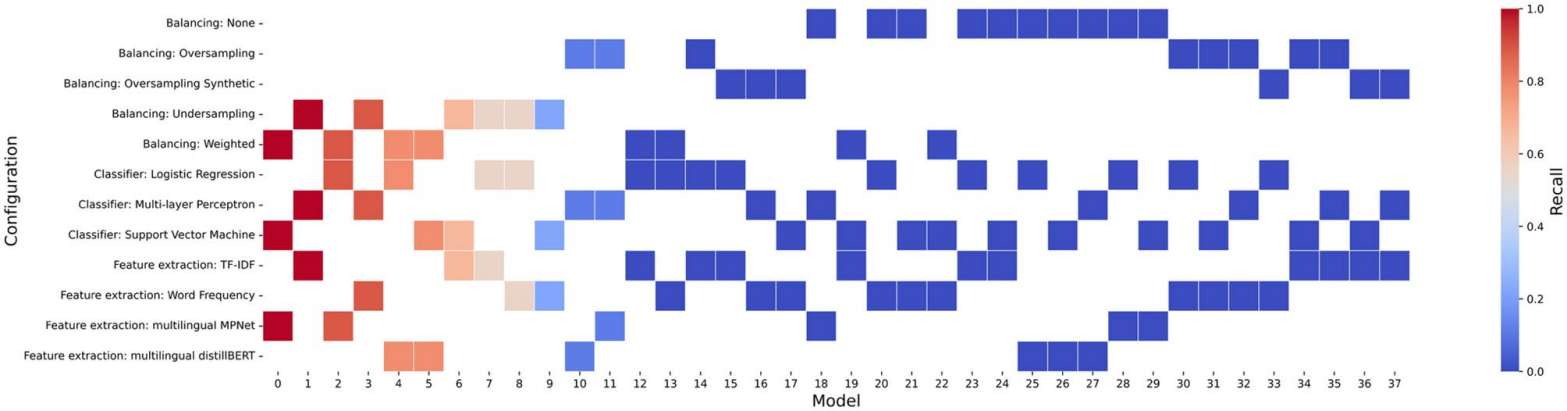
Heatmap depicting the configuration of 38 classification models tested in this study and their test set Recall. Columns on the x axis represent models (*n* = 38, also see Supp Mat Table 4 for the model number of each model) and rows on the y axis represent model configurations (i.e., model head, feature extractors, and balancing approach). Squares indicate the combination of the model head, feature extractor, and balancing approach used in each model, and colours depict the test set recall achieved by the model. Warmer colours (red) show higher recall and cooler colors (blue) show a lower recall. Additional performance scores are in Sup. Mat. Table 4. Notes: SVM parameters (weighted) : (class weight={0:0.50, 1: 63.06}, kernel:’linear’, probability = True, C = 0.01) Logistic Regression parameters (weighted): (class weight={0:0.50, 1: 63.06}, random state = 42, solver=’liblinear’, C = 0.01) MLP Classifier parameters: (activation=’logistic’, batch size = 16, hidden layer sizes=(), random state = 42) TF-IDF: term frequency inverse document frequency

The best performing classifier uses SVM, sentence-embedding using a multilingual pre-trained language model as the feature extractor, and weights to balance classes, achieving a test set recall of 100% and F1 of 0.071 (Fig. 2, Sup.Mat. Figure 1, Sup.Mat.Table 4). The classifier cut the manual labelling effort in a systematic synthesis by over 78% with a false positive error of < 22%. The high recall achieved indicates that the model effectively captures nearly all positive documents while minimizing false negatives (see Confusion Matrix in Sup. Mat. Table 2). Our ablation studies reveal that no single model component (i.e., encoding or weighting approach) contributes significantly more than the other to the overall architecture. This is evidenced by the fact that removing or replacing either of the two components results in a recall of 0% (Sup. Mat. Table 5). To test the performance of the best model, we trained the model using different train-test partitions and the results of these models did not deviate from those of the best model with the standard error of the model loss being less than 0.01 (Sup. Mat. Table 3). The second-best performing model, a multi-layer perceptron (MLP) classifier using TF-IDF with under-sampled training data also achieved a recall of 100%, but with a significantly lower F1 and precision (Sup. Mat. Table 4). As shown in Fig. 2, classifier performance strongly depends on the specific combination of model head, feature extractor and approach to data balancing. Further, our ablation studies reveal that no single model component (i.e., encoding or weighting approach) contributes significantly more than the other to the overall architecture. This is evidenced by the fact that removing or replacing either of the two components results in a recall of 0% (Sup. Mat. Table 5).

### Multilingual pre-trained models as encoders

Our results demonstrate the potential of multilingual pre-trained models for encoding a small corpus of Spanish-language text to train classification models (Fig. 2). Despite the challenge that only 44 documents in the entire dataset (0.79%) were relevant to biodiversity conservation, the pre-trained model (‘MPNet’) performed well in capturing contextual information for each class. However, the sentence embedding alone can’t achieve high performance and it needs to be used with appropriate weighting and classification head too.

### Dealing with imbalanced data

We found that weighting the loss function was the most effective strategy for addressing extreme class imbalance, a common challenge in evidence synthesis tasks and literature classification (Fig. 2). By assigning higher importance to underrepresented classes, this approach improves the model’s sensitivity to rare but relevant documents, ensuring better recall without compromising precision. The MLP classifier also achieved a comparable recall by undersampling training data. However, reducing the training data may result in losing valuable information, potentially leading to a skewed understanding of the underlying linguistic patterns and biasing model predictions and limiting generalizability.

### Prediction explanations

**Fig. 3:**
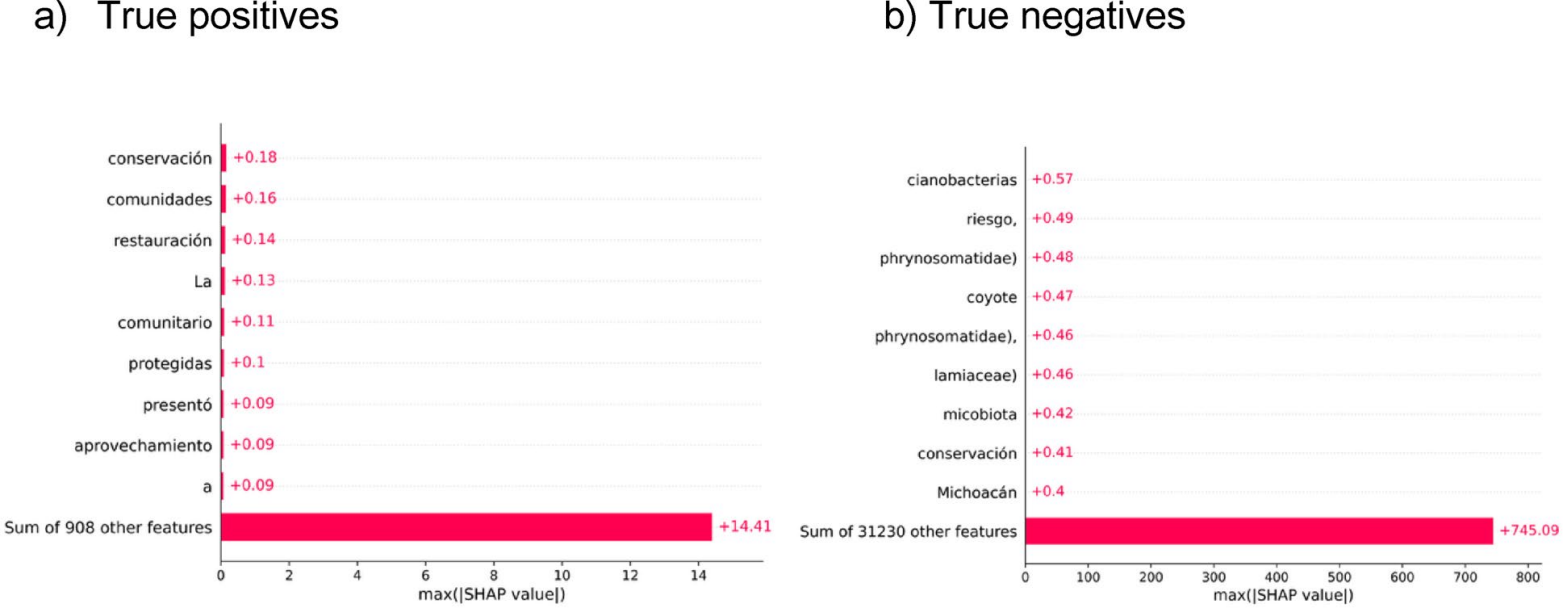
Summary of the words having the largest impact to increase any true (**a**) positive and (**b**) negative predictions, if present. The x axis shows the max absolute SHAP value expressed as the SVM decision scores. The bar at the bottom of the figure represents the sum of all other words in the text. Translation to English of the words in the (**a**) positive prediction are: *conservación* = conservation, *comunidades* = communities, *restauración =* restoration, *La* = the, *comunitario =* community, *protegidas =* protected, *presentó=*presented, *aprovechamiento = utilization*,* a =* to; and (b) negative predictions are *cianobacterias* = cyanobacteria, *riesgo* = risk, phrynosomatidae = phrynosomatidae, *coyote* = coyote, lamiaceae = lamiaceae, “*microbiota* = microbiota, *conservación* = conservation, and Michoacán = Michoacán

Using SHAP, we measured the role of each word or set of words in the classifier’s predictions –note that because the best-preforming classifier used sentence embeddings as input features, stop words are also included in this analysis. SHAP values explain the change in the model’s prediction when a word is included versus when it’s not. Thus, the SHAP value that a word gets depends on their context and it is not related to high word occurrence, but on the relative importance that word has in the instance. Figure 3 shows a summary of the words having the largest impact in any instance based on the max absolute SHAP value. The words in Spanish and their translations to English that had the largest impact in predicting a positive instances are “*conservación* = conservation”, “*comunidades* = communities”, “*restauración =* restoration”, “*La =* the”, “*comunitario =* community”, “*protegidas =* protected”, “*presentó=*presented”, “*aprovechamiento =* utilization*”*,* “a =* to”, many of them being strong indicators of the corpus domain, biodiversity conservation actions, particularly conservation interventions and their consequences. Words from models trained on different train-test partitions follow the same pattern, for instance *“parques =* parks”, “*restauración* = restoration”, “*fuego* = fire” (Sup. Mat. Figure 2). The words in Spanish and their translations to English that had the largest impact in predicting negative instances are “*cianobacterias* = cyanobacteria”, “*riesgo* = risk”, “phrynosomatidae = phrynosomatidae”, “*coyote* = coyote”, “lamiaceae = lamiaceae”, “*microbiota* = microbiota, *conservación* = conservation”, and ‘Michoacán = Michoacán” (Fig. 3). The word *conservation* appears as an important word in both true negative and true positive documents (Fig. 3). The reason for this can be because words in embedding models do not have a fixed directional impact (positive or negative). Instead, their impact is contextual: the embedding model encodes a word’s meaning based on its surroundings (e.g., ‘wildlife conservation’ vs. ‘energy conservation’), and SHAP aggregates these local effects. Thus, the same word can push predictions in different directions depending on its usage in the text.

**Fig. 4:**
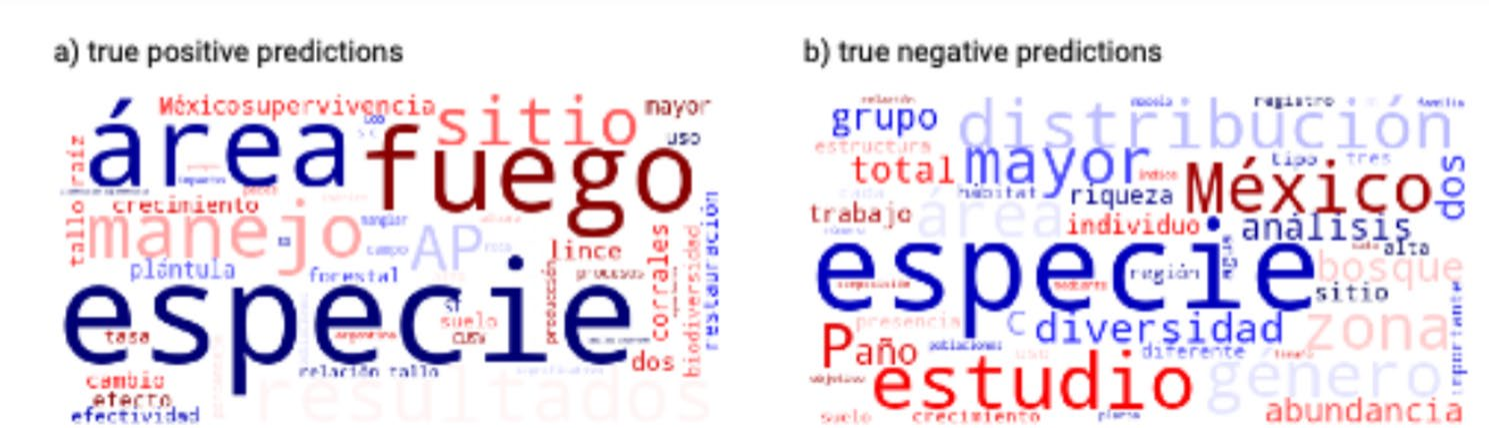
Word clouds of frequently used words in (**a**) true positive and (**b**) true negative predictions. The top five most frequently used words in (**a**) are *especie = species*, followed by *área = area*, *fuego = fire*, *manejo = management* and in (**b**) *especie = species*, *estudio = study*, *México =* Mexico, *bosque = forest*,* distribución = distribution*,* género = genus/gender*

Perhaps not surprising, the words having the largest impact to increase a true positive and negative prediction did not match the most frequently used words in the true positive nor negative predicted class (Fig. 4). It often happens that the most frequent words carry little class-specific information, this being the reason why TF-IDF is often better than TF when extracting words using word-based approaches. The top five words in the true positives are *especie =* species (normalized frequency of 100%), *fuego =* fire and *área =* area (normalized frequency of 60%) and *resultados =* results *=* and *manejo =* management (normalized frequency of 50%) (see Calculating normalized frequency scores in Sup. Mat. and normalized frequency of words in https://figshare.com/s/0fa0886ef5734a77893c*).* Similarly, the normalized frequencies of the top five words in the predicted true negatives show that *especie =* species is the most frequent word followed by *estudio =* study occurring 28% as often as the most common word, and *México =* Mexico, *bosque =* forest, *distribución =* distribution, and *género =* genus/gender appear 20% as often as the most common word.

**Fig. 5:**
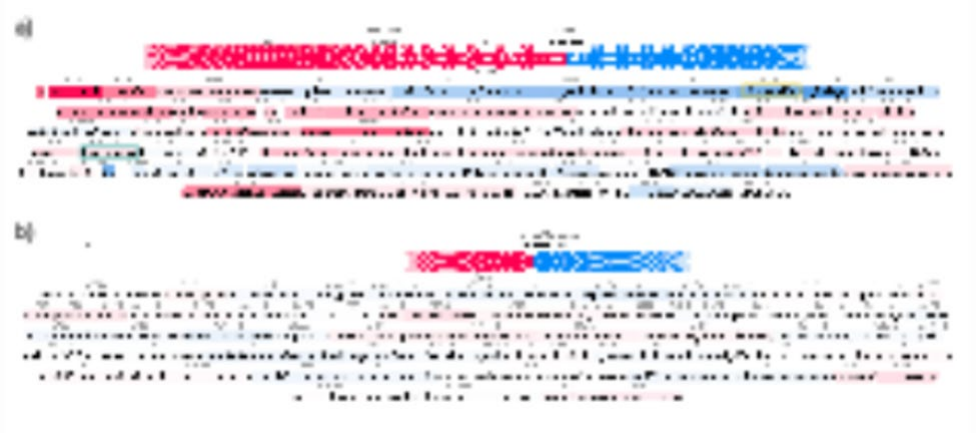
Example of the SHAP contributions (decision score) on the model’s output of correctly classified (**a**) positive and (**b**) negative documents. The *base value* is the baseline value that the model outputs when no specific input words are considered. It acts as a reference point to explain how much each word pushes the prediction higher or lower relative to this baseline. The *f(inputs)* is the sum of all SHAP values output of the model for the full original input. Each word’s SHAP value is above each word/group of words, and they represent the contribution of that specific word to the change in the model’s output (decision score) compared to the base value. Words in pink/red push the model’s prediction towards a higher decision score value for the predicted class whilst words in blue model’s prediction towards a lower decision score value for the predicted class. The intensity of the colour on the text indicates the magnitude of the impact (i.e., strength of the words contribution)

The word contributions to the model prediction in a correctly classified positive and negative document are shown in Fig. 5 as an example (See full length of predictions in https://figshare.com/s/89df965ebf96e62bf1e3*).* Words in red increase the SVM decision scores and thus the positive predicted probability while words in blue increase the predicted negative probability. The *f(inputs)* value is the sum of all word contributions, which added up to 0.367 compared to the base value in Fig. 5a, making it a positive prediction. Following the same logic, the *f(inputs)* value in Fig. 5b is -0.692, showing the word contributions to a negative class prediction. In the document shown in Fig. 5a, the top two words that contributed to the positive prediction example are highly relevant to biodiversity conservation actions, for instance*“conservación =* conservation*”*,* “reserva =* reserve (noun)*”*,* and “mamíferos =* mammals”. Conversely, words like “*tropica = tropical”* and *“biodiversidad =* biodiversity*”* decreased the probability. Interestingly, in this document the word “*biodiversidad”* contributes both positively (SVM decision score = 0.023, in a green square) and negatively (SVM decision score=-0.115, in an orange square) to the model’s output as highlighted in Fig. 5a. This shows the nature of embedding models that encode not just the word itself but its interaction with surrounding words capturing nuanced semantic information from contextual relationships. For instance, the word “biodiversity*”* (in Spanish biodiversidad) in a green square in Fig. 5a, is close to the word “to preserve” (in Spanish conservar) potentially influencing the importance of the word biodiversity.

### Error analysis

**Fig. 6:**
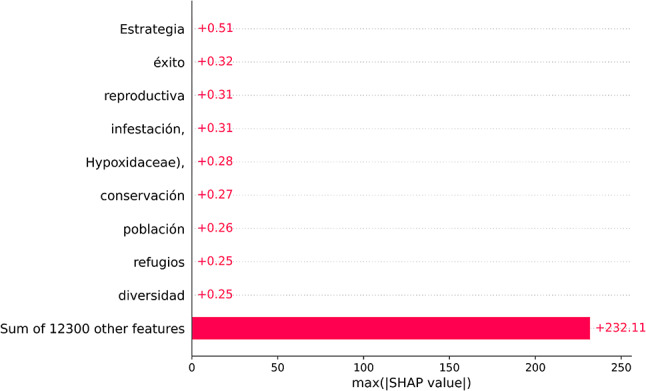
Summary of the words having the largest impact to increase any false positive predictions, if present. The x axis shows the SHAP values expressed as SVM decision scores and are calculated on the max absolute SHAP value. The bar at the bottom of the figure represents the sum of all other words in the text. Translation to English of the words are: “*estrategia =* strategy”, “*éxito =* success”, “*reproductiva =* reproductive”, “*infestation =* infestation”, “*Hypoxidaceae* = Hypoxidaceae”, “*conservación* = conservation”, “*población* = population”, “*refugio* = refuge”, “*diversidad =* diversity”

To understand why the model misclassified a positive document we calculated the SHAP scores of the false negative predictions (Fig. 6, Sup. Mat. Figure 2, See full length of predictions in https://figshare.com/s/74145a9cf53329b69bd7*).* Translations of the top words that strongly push negative documents to be classified as positives are shown in Fig. 6. Several of these words are words associated with conservation interventions, the positive class domain including “*estrategia =* strategy”, “*éxito =* success”, “*conservación* = conservation”, “*refugio* = refuge”, “*diversidad =* diversity”. We suspect that the presence of these words “confused” the classifier into predicting 234 false positives. Words from models trained on different train-test partitions follow the same pattern, for instance “*conservación* = conservation”, “*reforestación* = reforestation”, “*sostenibilidad =* sustainability”, “*resiliencia =* resilience” (Sup. Mat. Figure 2). Furthermore, we suspect that there might be some model bias when learning features in the negative class as we see that the model overweights words like *“estrategia”*, *“éxito”*, “*infestación*”, and others (SHAP values = 0.51, 0.32, and 0.31 and so on) strongly associating these to the positive class. Another bias could be from the negative documents, in which those terms are absent, rare or surrounded by words with strong conservation semantic meaning.

## Discussion

Using supervised machine learning we developed a Spanish-language text classifier to identify relevant scientific documents on the effectiveness of biodiversity conservation actions. A key finding of our study is the robustness of the model architecture combining transformer-based multilingual models to represent semantic sentence-level features and weighted loss function to deal with a highly imbalanced dataset. The sentence embedding model contributed to achieving strong classification outcomes, very likely because it learned deep representation of the words by pre-training on contextual representation using a large corpus with bidirectionality, whereas the traditional models use frequency-based feature extractors. The encoder’s capability to handle the complexities of non-English linguistic structures is an advantage for multilingual text applications. The use of SHAP [48] further enhanced explainability by providing insights into how the model generates predictions and showed that words with deep semantic meaning to biodiversity conservation interventions, the domain of the positive class, were the words with largest importance. Such transparency is crucial for fostering trust in automated classification systems, as it allows researchers to understand not only what the model predicts but also why and how those predictions are made. This capability makes our approach a valuable tool for automated non-English-language text classification applications in global conservation research.

Our work demonstrates that using transformer-based multilingual models to encode non-English-language text at the sentence level (e.g., SBERT), combined with a simple classification head like logistic regression can also yield a lightweight yet effective multilingual classifier. A classification algorithm with a logistic regression head trained only on English-language biodiversity data has also shown exceptional performance [16, 38, 49]. Similarly, research testing sentence-level representations of English-language text against token-based ones have shown the robustness of the former [49]. Furthermore, a common issue in classification problems are imbalanced datasets [15, 50, 51], and we addressed this issue by weighting the loss function to balance classes. An approach that combines these methodologies is valuable for the screening stage in environmental evidence synthesis, where language barriers often limit access to evidence produced in highly biodiverse regions where English is not widely spoken. However, because our training data spans from 1992 to 2019, our model might need to be updated using adaptive text classification frameworks to keep up with the fluidity of scientific language where a temporal and conceptual drift exists (i.e., new/modern concepts, terminology, and definitions).

Alternative classification methodologies—such as XLM-Roberta models, virtual agents, and other generative or reasoning AI systems—may result in similar classification performance and aid the screening stage in evidence syntheses. These alternative approaches, either independently or in combination with our methods, could enhance overall classification outcomes. For instance, screening data for a systematic review on electric vehicles using GPT-4 achieved comparable results to our best-performing classifier [52]. More work should examine the advantages of these methodologies to determine the most effective strategies for English and non-English-language text classification in research.

Another avenue for future exploration involves leveraging the multilingual embeddings used in this study and assessing the model’s ability to generalize across other non-English languages. In this same line, future work can perform zero-shot learning, where our model is retrained using a different non-Spanish Latin language with very little labelled data, for instance the Portuguese language. The knowledge transferred from our model can help bootstrap the model’s performance in the target language. Such a model could possibly perform as good as our best performing model. Expanding the model’s application beyond Spanish-language texts could provide valuable insights into its versatility and potential for broader evidence synthesis. Additionally, future research should evaluate whether pre-trained models with extended sequence lengths or using document embedding can generate improved embeddings, leading to better classification performance (i.e., F1 score). Furthermore, fine-tuning our models could achieve better precision, a crucial factor in, e.g., health-related syntheses, where comprehensive and precise coverage is essential [13, 14]. As a result, these classifiers would enable fast and complete identification of relevant literature for biodiversity conservation evidence syntheses. However, we caution that our approach is only for one step in the evidence syntheses process and either additional AI algorithms should be validated and included in the pipeline or fluent speakers of a language are needed to extract data and assess publications.

Box 2 - Practical recommendations to leveraging pre-trained language models. For researchers and practitioners interested in leveraging pre-trained language models for Spanish text classification we offer practical recommendations:

- Multilingual vs. monolingual models: while multilingual models (e.g., *MPNet*) perform well across languages, monolingual Spanish models can also achieve good results for Spanish-specific tasks due to specialized vocabulary and training data. To select the most appropriate model, check the HuggingFace leaderboard of pre-trained multilingual models (https://huggingface.co/spaces/mteb/leaderboard) and always consider the domain in which the model has been trained –this can have a huge impact in how the model “understands” your text. Also, match your text preprocessing to the model’s training regime (i.e., casing, tokenization, maximum sequence length).
- Explainable AI (a.k.a. XAI): consider including techniques to understand why your model makes a certain prediction, rather than treating it as a “black box”. Tools like SHAP (SHapley Additive exPlanations) [48] can visually highlight which specific words or phrases in the text most influenced the classification outcome. These visualisations increase transparency and can help you understand model errors and biases.
- Computational resources: consider a language model size that matches your computational capabilities. The HuggingFace model leaderboard (https://huggingface.co/spaces/mteb/leaderboard) offers information on model size. If a big model is required, free cloud computing services, like Google Collab are available.

## Conclusions

Our study shows that integrating multilingual pre-trained models for text encoding, a weighted loss function for class balancing, and support vector machine as the classification algorithm enables a classifier to perform effectively on Spanish-language text. Multilingual text embeddings allow learning more accurate classifiers without large amounts of non-English labelled data expanding the scope of knowledge covered in an evidence synthesis by including non-English language evidence. Furthermore, non-English-language text classifiers can streamline the screening of titles and abstracts, accelerating the identification of relevant documents in conservation science. Automating and making this step of the synthesis process multilingual not only improves efficiency but also allows researchers to focus on analyzing high-relevance documents and ensuring broader coverage of non-English-language evidence in environmental evidence syntheses.

## Supplementary Information

Below is the link to the electronic supplementary material.

Supplementary Material 1.

## Data Availability

The datasets generated and/or analysed during the current study and all codes used in the analysis are available at: https://github.com/vberdejoespinola/translate-text-classifier-spanish . This repository contains an overview of the scripts, the data needed to run each script, and a list of the packages used.
