# Supplementary material for: Spanish-language text classification for environmental evidence synthesis using multilingual pre-trained models
Source: Environ Evid. 2025 Nov 12;14:21. doi: 10.1186/s13750-025-00370-9 (PMC12613578; doi:10.1186/s13750-025-00370-9)

**Spanish-language text classification for conservation evidence synthesis using multi-lingual pre-trained models**

**Supplementary Material**

*Table 1. List of journals used as training data.*

| **Journal title in Spanish** | **Country** | **Number of papers screened in**[**^1^**](https://www.zotero.org/google-docs/?cDFaIQ) | **Years screened first in**[**^1^**](https://www.zotero.org/google-docs/?NG2xPz) | **Years screened last in**[**^1^**](https://www.zotero.org/google-docs/?IfFrBf) | **Total relevant documents** |
| --- | --- | --- | --- | --- | --- |
| Acta Zoológica Mexicana | Mexico | 1195 | 1984 | 2019 | 0 |
| Barbastella | Spain | No record | 2012 | 2019 | 4 |
| Ecologia Aplicada | Peru | 329 | 2002 | 2018 | 1 |
| Ecología Austral | Argentina | 449 | 2001 | 2019 | 1 |
| Huitzil | Mexico | 275 | 2000 | 2019 | 1 |
| Galemys | Spain | No record | 1997 | 2017 | 9 |
| Madera y Bosques | Mexico | 485 | 2005 | 2019 | 2 |
| Mastozoología Neotropical | Argentina | 662 | 1994 | 2018 | 7 |
| Quebracho | Argentina | 144 | 2007 | 2018 | 2 |
| Revista Chilena de Historia Natural | Chile | 3131 | 1987 | 2019 | 4 |
| Revista de Biologia Tropical | Costa Rica | 4152 | 1989 | 2019 | 1 |
| Revista Mexicana de Biodiversidad | Mexico | 1593 | 2005 | 2019 | 6 |
| Revista Mexicana de Ciencias Forestales | Mexico | 455 | 2011 | 2018 | 4 |
| Therya | Mexico | 376 | 2010 | 2019 | 2 |

*Table 2. a) Train and b) Test set confusion matrices of best performing model. Rows represent actual (true) categories and columns represent predicted categories. Each cell indicates how many instances of a true category (row) were classified into a predicted category (column).*

1. Train set confusion matrix

|  | **Predicted Negative** | **Predicted Positive** |
| --- | --- | --- |
| **True Negative** | 3538 | 867 |
| **True Positive** | 0 | 35 |

1. Test set confusion matrix

|  | **Predicted Negative** | **Predicted Positive** |
| --- | --- | --- |
| **True Negative** | 867 | 234 |
| **True Positive** | 0 | 9 |

*Formula for best performing model*

$$SVC(kernel='linear', class\_weight=\{1:63.06, 0:0.50\}, probability=True, C=0.01)$$

*Formula for class weights*

$Weight for class 0= len(all instances)/(len(negative instances)*2)$ *= 0,50*

$Weight for class 1= len(all instances)/(len(positive instances)*2)$ *= 63.06*

*Table 3. Test set scores and training loss of the best performing model when training the model with different train-test partitions (random initialisations=12, 24, 36, 42). Models are SVM with class weights, using a ‘liblinear’ solver, and a C regularisation parameter of 0.01.*

| **Model seed (x)** | **F1** | **Precision** | **Recall** | **Log_loss** |
| --- | --- | --- | --- | --- |
| 12 | 0.062 | 0.032 | 0.889 | 0.0381 |
| 24 | 0.058 | 0.03 | 0.889 | 0.0367 |
| 36 | 0.061 | 0.032 | 1.0 | 0.0336 |
| 42 | 0.071 | 0.037 | 1.0 | 0.0369 |

*Standard error of log loss: 0.00096*


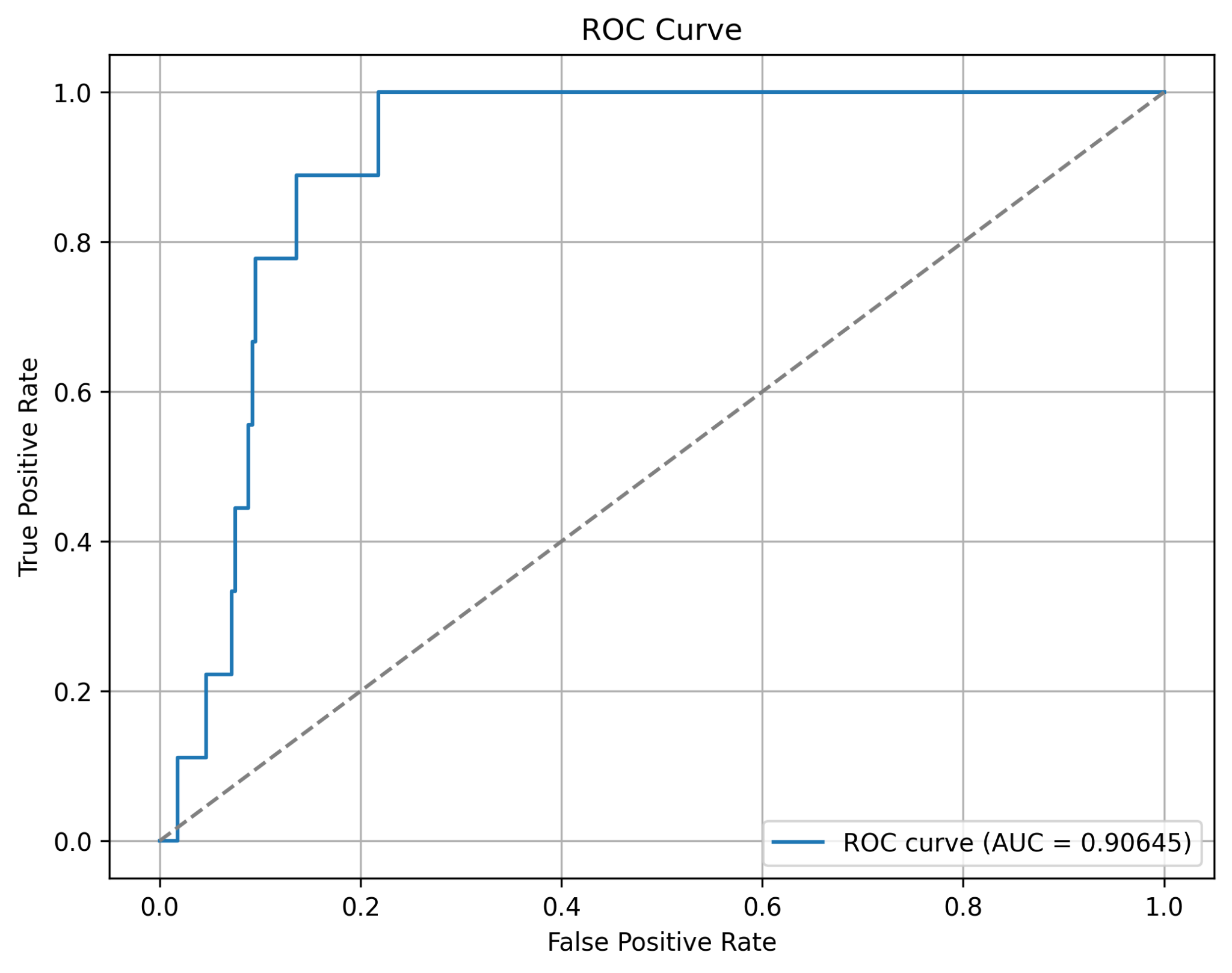


*Figure 1. Test set ROC-AUC scores of the best performing model: a support vector machine, with class weights and text encoded with sentence embeddings.*

*Calculating feature normalized frequency scores*

The normalized frequency​ of a feature is calculated based on the feature’s frequency in the text in the predicted class, adjusted by frequency of the most frequent word (which is scaled to 1.0). The most frequent word in the text gets a normalized value of **1.0**. All other words are scaled proportionally relative to this maximum frequency. It is computed as:

n_i_ = f_i_/ f_max_

Where:

f_ii_ is the absolute frequency (count) of the word i,
f_max_ is the absolute frequency of the most frequent word,

*Table 4. Test set scores of all models trained. Formulas are shown in the notes below the table.*

| **Model number** | **Model head** | **Feature extraction** | **Weighting** | **F1** | **Precision** | **Recall** |
| --- | --- | --- | --- | --- | --- | --- |
| 0 | SVC | paraphrase-multilingual-mpnet-base-v2 | Weighted | 0.071 | 0.037 | 1 |
| 1 | Multi-layer Perceptron | TF-IDF | Undersampling | 0.05 | 0.026 | 1 |
| 2 | Logistic Regression | paraphrase-multilingual-mpnet-base-v2 | Weighted | 0.071 | 0.037 | 0.889 |
| 3 | Multi-layer Perceptron | Word count | Undersampling | 0.056 | 0.029 | 0.889 |
| 4 | LogisticRegression | distiluse-base-multilingual-cased-v1 | Weighted | 0.096 | 0.051 | 0.778 |
| 5 | SVC | distiluse-base-multilingual-cased-v1 | Weighted | 0.089 | 0.047 | 0.778 |
| 6 | SVC | TF-IDF | Undersampling | 0.074 | 0.039 | 0.667 |
| 7 | Logistic Regression | TF-IDF | Undersampling | 0.058 | 0.031 | 0.556 |
| 8 | Logistic Regression | Word count | Undersampling | 0.04 | 0.021 | 0.556 |
| 9 | SVC | Word count | Undersampling | 0.024 | 0.012 | 0.222 |
| 10 | Multi-layer Perceptron | distiluse-base-multilingual-cased-v1 | Oversampling | 0.083 | 0.067 | 0.111 |
| 11 | Multi-layer Perceptron | paraphrase-multilingual-mpnet-base-v2 | Oversampling | 0.062 | 0.043 | 0.111 |
| 12 | Logistic Regression | TF-IDF | Weighted | 0 | 0 | 0 |
| 13 | Logistic Regression | Word count | Weighted | 0 | 0 | 0 |
| 14 | Logistic Regression | TF-IDF | Oversampling | 0 | 0 | 0 |
| 15 | Logistic Regression | TF-IDF | Synthetic Oversampling | 0 | 0 | 0 |
| 16 | Multi-layer Perceptron | Word count | Synthetic Oversampling | 0 | 0 | 0 |
| 17 | SVC | Word count | Synthetic Oversampling | 0 | 0 | 0 |
| 18 | Multi-layer Perceptron | paraphrase-multilingual-mpnet-base-v2 | None | 0 | 0 | 0 |
| 19 | SVC | TF-IDF | Weighted | 0 | 0 | 0 |
| 20 | Logistic Regression | Word count | None | 0 | 0 | 0 |
| 21 | SVC | Word count | None | 0 | 0 | 0 |
| 22 | SVC | Word count | Weighted | 0 | 0 | 0 |
| 23 | Logistic Regression | TF-IDF | None | 0 | 0 | 0 |
| 24 | SVC | TF-IDF | None | 0 | 0 | 0 |
| 25 | Logistic Regression | distiluse-base-multilingual-cased-v1 | None | 0 | 0 | 0 |
| 26 | SVC | distiluse-base-multilingual-cased-v1 | None | 0 | 0 | 0 |
| 27 | Multi-layer Perceptron | distiluse-base-multilingual-cased-v1 | None | 0 | 0 | 0 |
| 28 | Logistic Regression | paraphrase-multilingual-mpnet-base-v2 | None | 0 | 0 | 0 |
| 29 | SVC | paraphrase-multilingual-mpnet-base-v2 | None | 0 | 0 | 0 |
| 30 | Logistic Regression | Word count | Oversampling | 0 | 0 | 0 |
| 31 | SVC | Word count | Oversampling | 0 | 0 | 0 |
| 32 | Multi-layer Perceptron | Word count | Oversampling | 0 | 0 | 0 |
| 33 | Logistic Regression | Word count | Synthetic Oversampling | 0 | 0 | 0 |
| 34 | SVC | TF-IDF | Oversampling | 0 | 0 | 0 |
| 35 | Multi-layer Perceptron | TF-IDF | Oversampling | 0 | 0 | 0 |
| 36 | SVC | TF-IDF | Synthetic Oversampling | 0 | 0 | 0 |
| 37 | Multi-layer Perceptron | TF-IDF | Synthetic Oversampling | 0 | 0 | 0 |

Notes:

SVM parameters: (kernel:’linear’, probability=True)

SVM parameters (weighted): (class weight={0:0.50, 1: 63.06}, kernel:’linear’, probability=True, C=0.01)

Logistic Regression parameters: (random state=42, solver='liblinear')

Logistic Regression parameters (weighted): (class weight={0:0.50, 1: 63.06}, random state=42, solver='liblinear', C=0.01)

Multi-layer Perceptron parameters: (activation='logistic', batch size=16, hidden layer sizes=(), random state=42)

TF-IDF: term frequency inverse document frequency

Table 5. Test set scores of models run for the ablation study.

| **Model head** | **Feature extraction** | **Weighting** | **F1** | **Precision** | **Recall** |
| --- | --- | --- | --- | --- | --- |
| SVC | paraphrase-multilingual-mpnet-base-v2 | None | 0 | 0 | 0 |
| SVC | TF-IDF | Weighted | 0 | 0 | 0 |
| SVC | Word count | Weighted | 0 | 0 | 0 |
| SVC | paraphrase-multilingual-mpnet-base-v2 | Weighted | 0.071 | 0.037 | 1 |

Notes:

SVM parameters: (kernel:’linear’, probability=True)

SVM parameters (weighted): (class weight={0:0.50, 1: 63.06}, kernel:’linear’, probability=True, C=0.0

*Figure 2. Summary of the features having the largest impact to increase true positive, true negative and false positive predictions of models trained with different train-test partitions (random initialisations=12, 24, 36).*

***Random initialisation 12***

True positives True negatives False positives


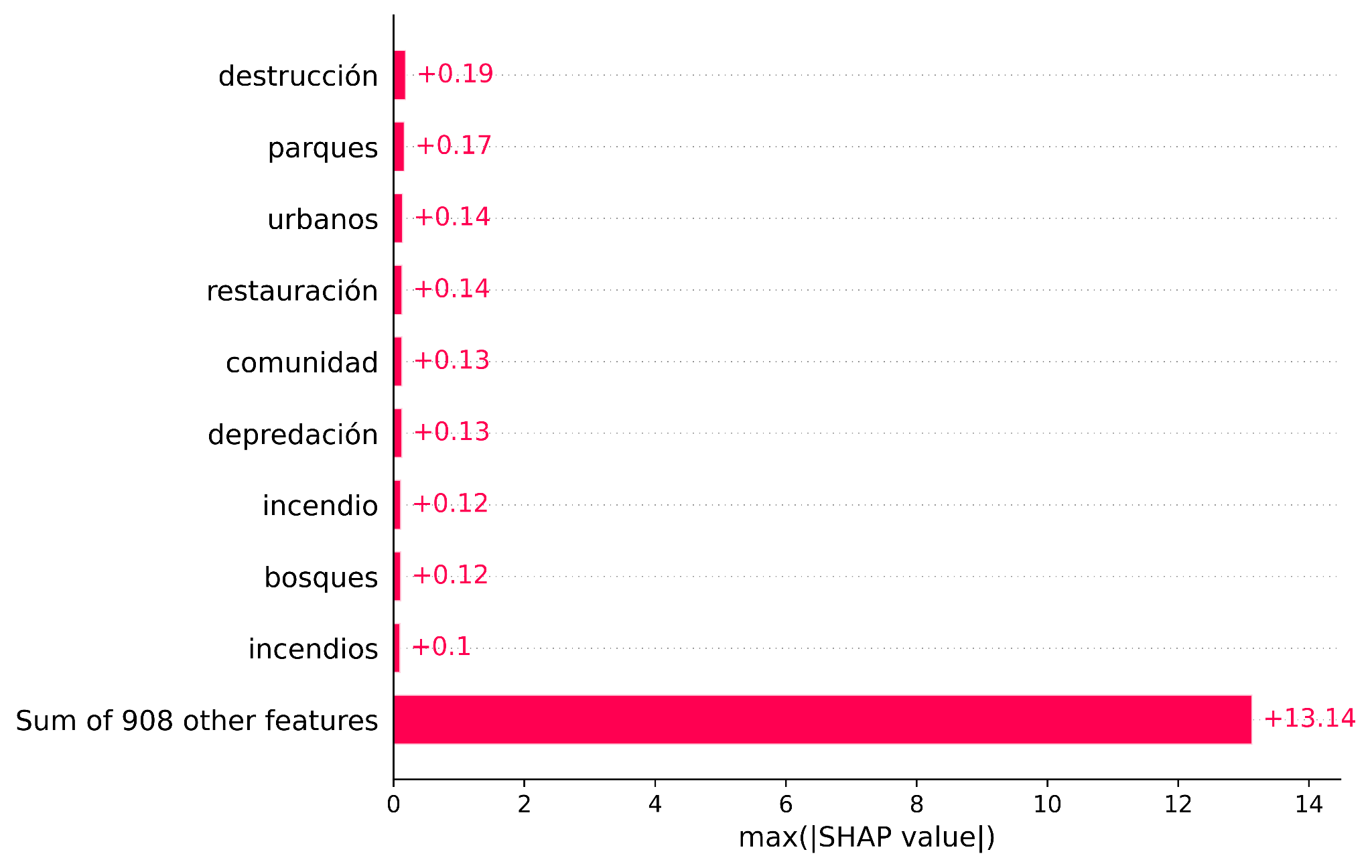

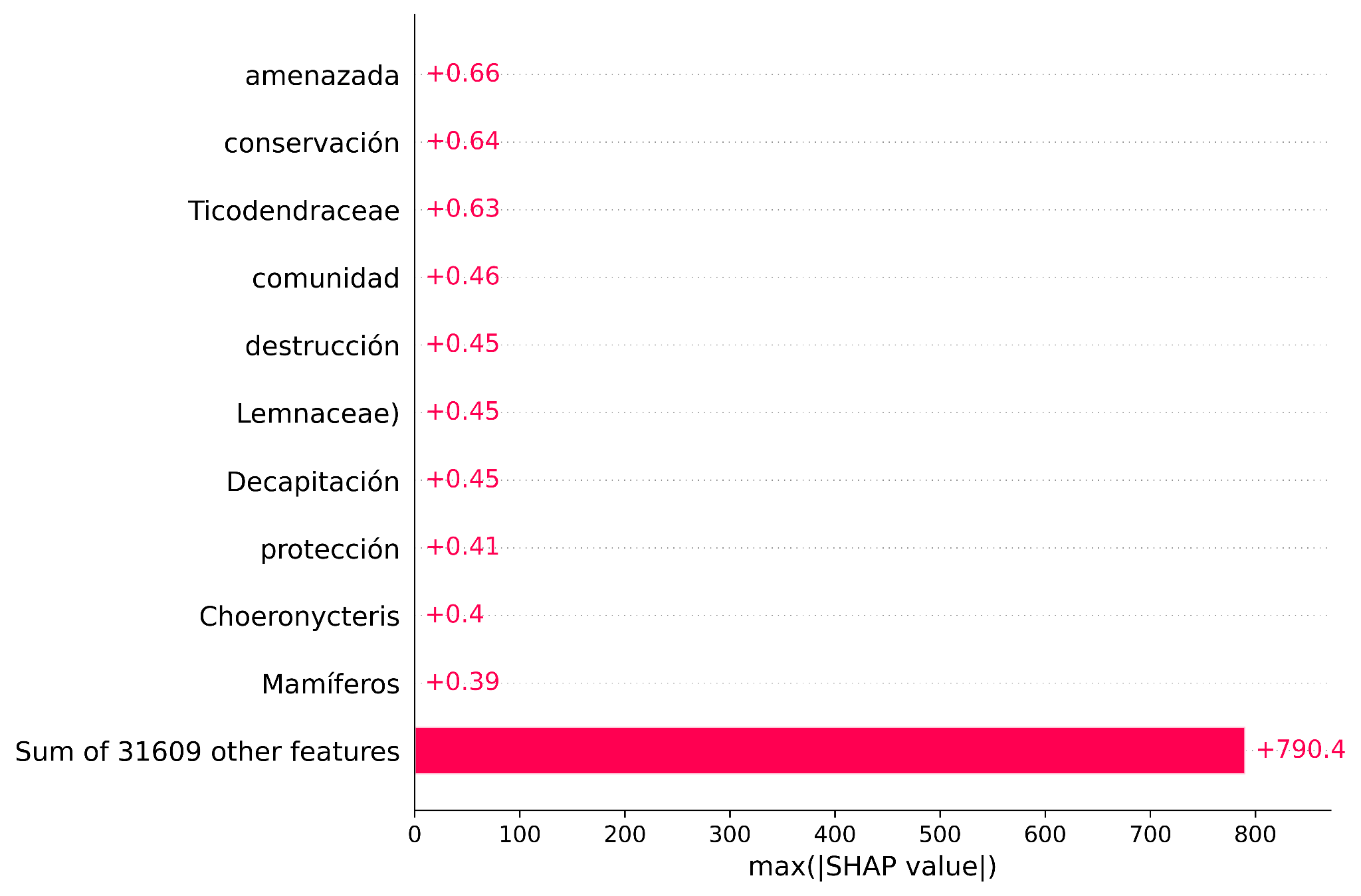

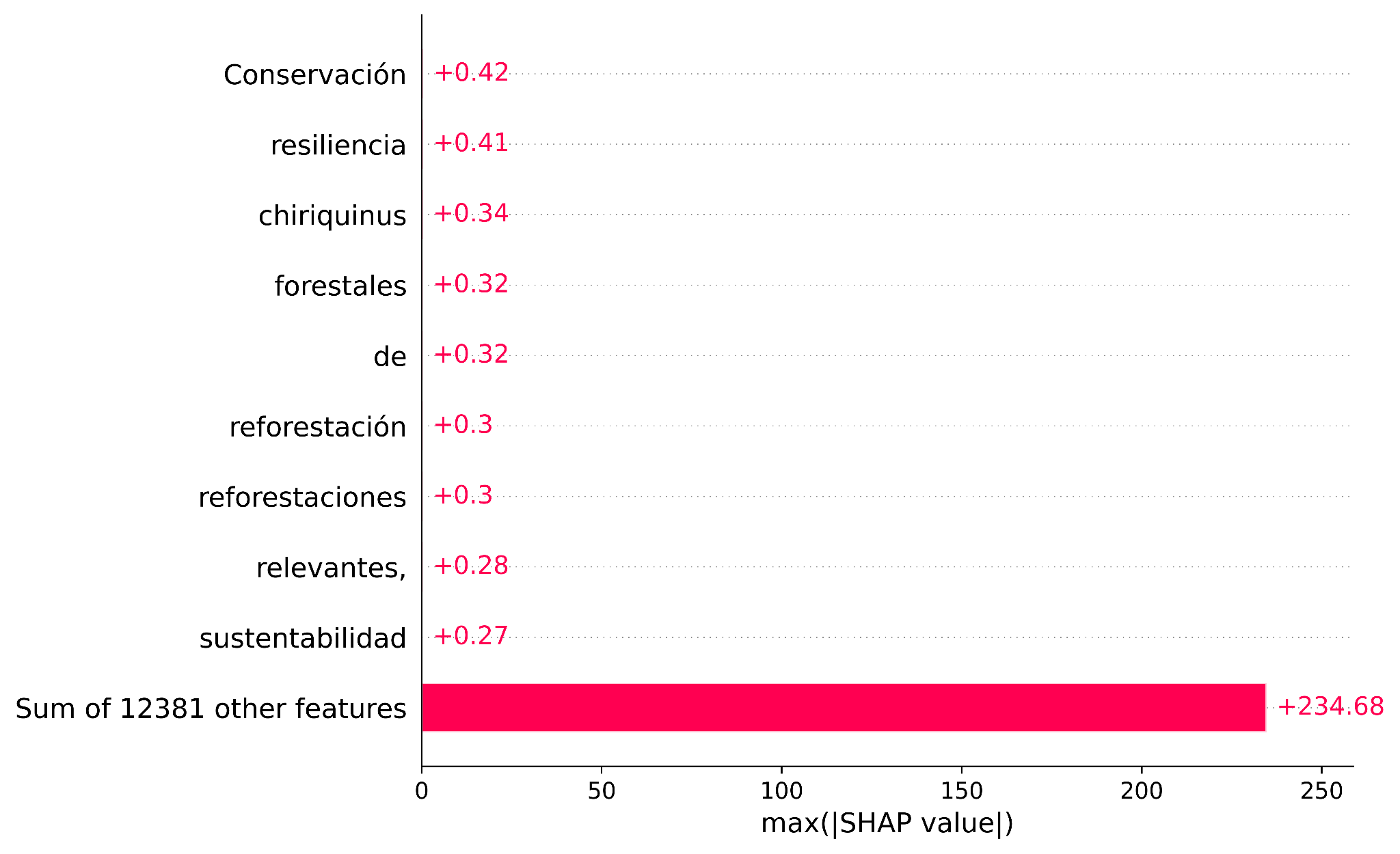


***Random initialisation 24***

True positives True negatives False positives


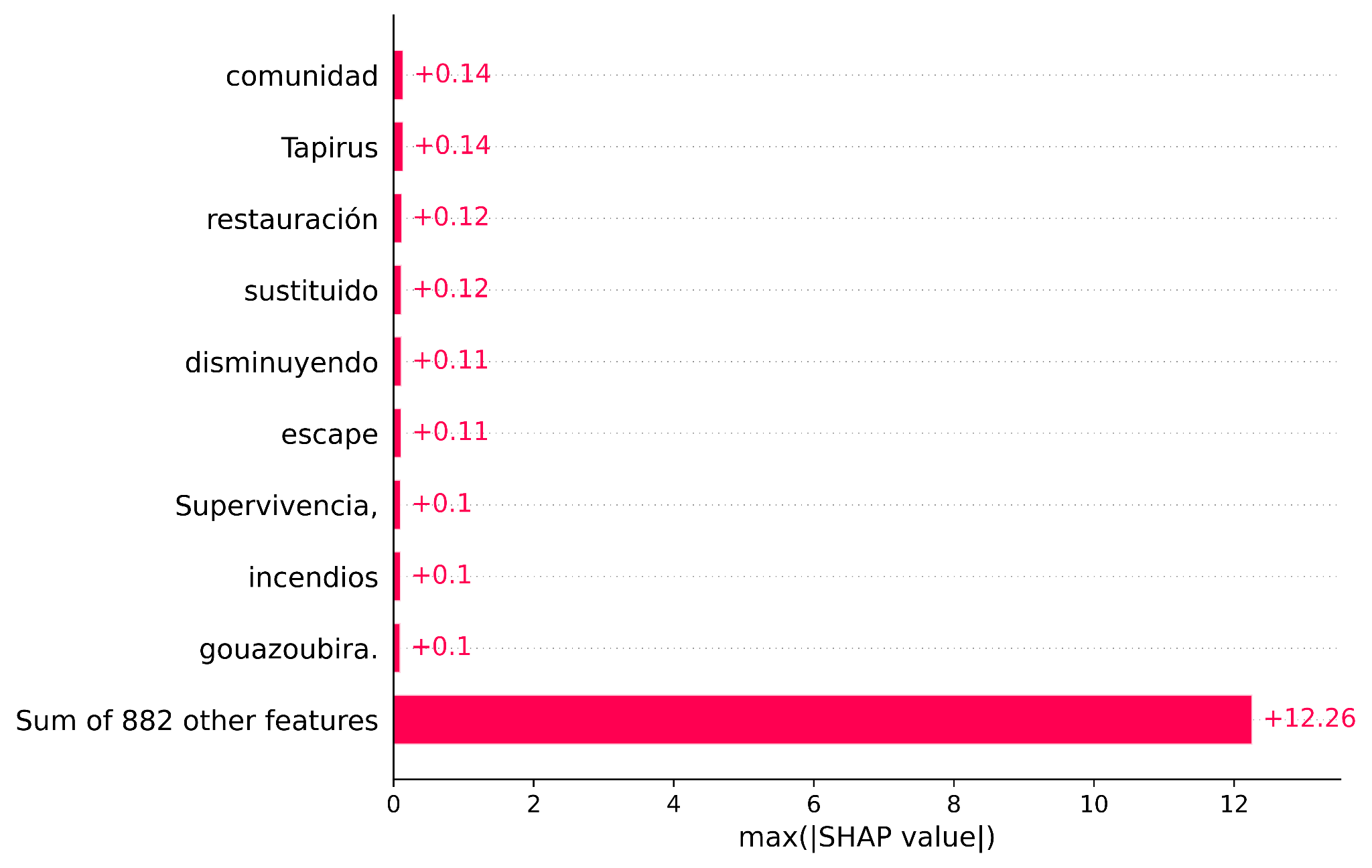

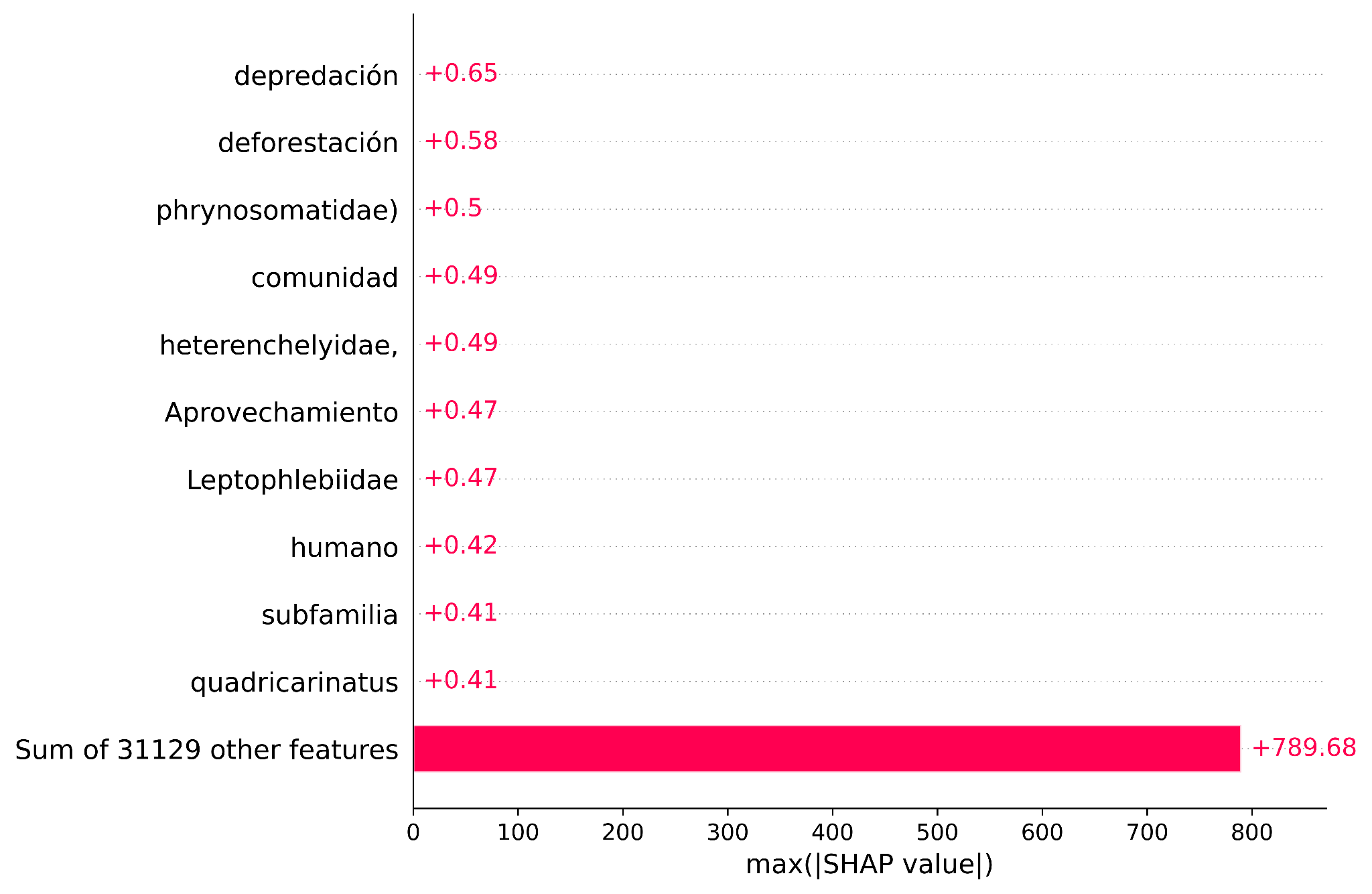

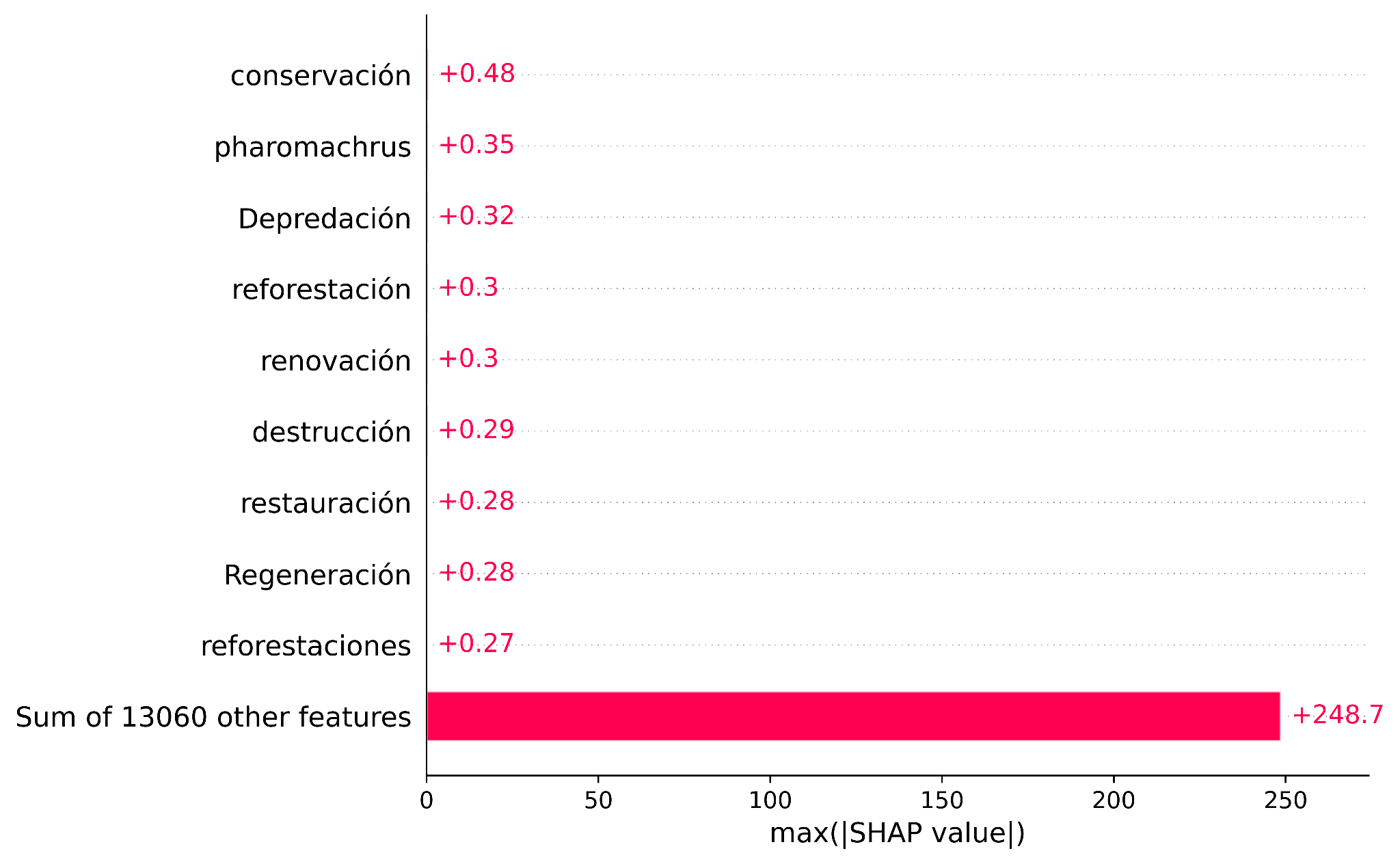


**Random initialisation 36**

True positives True negatives False positives


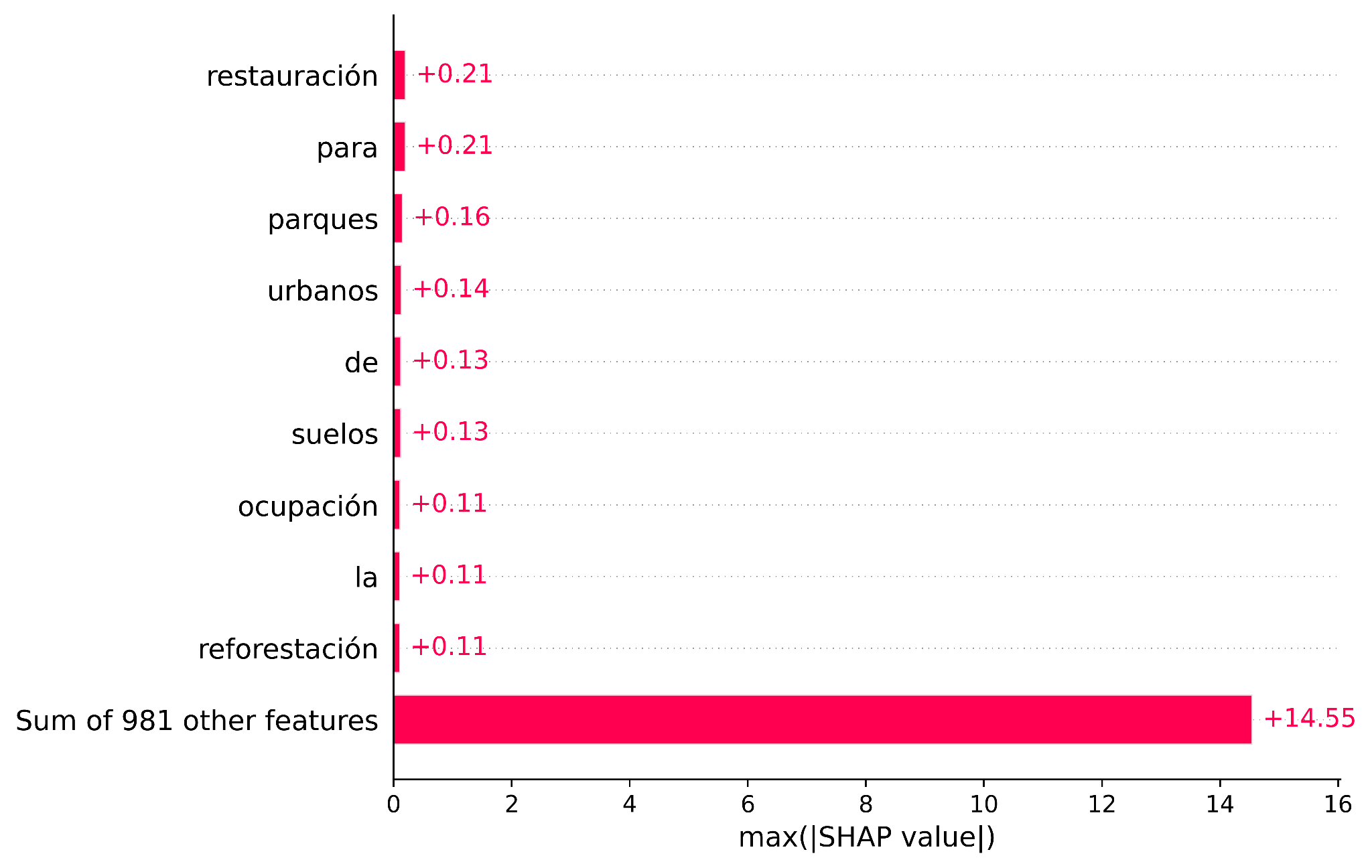

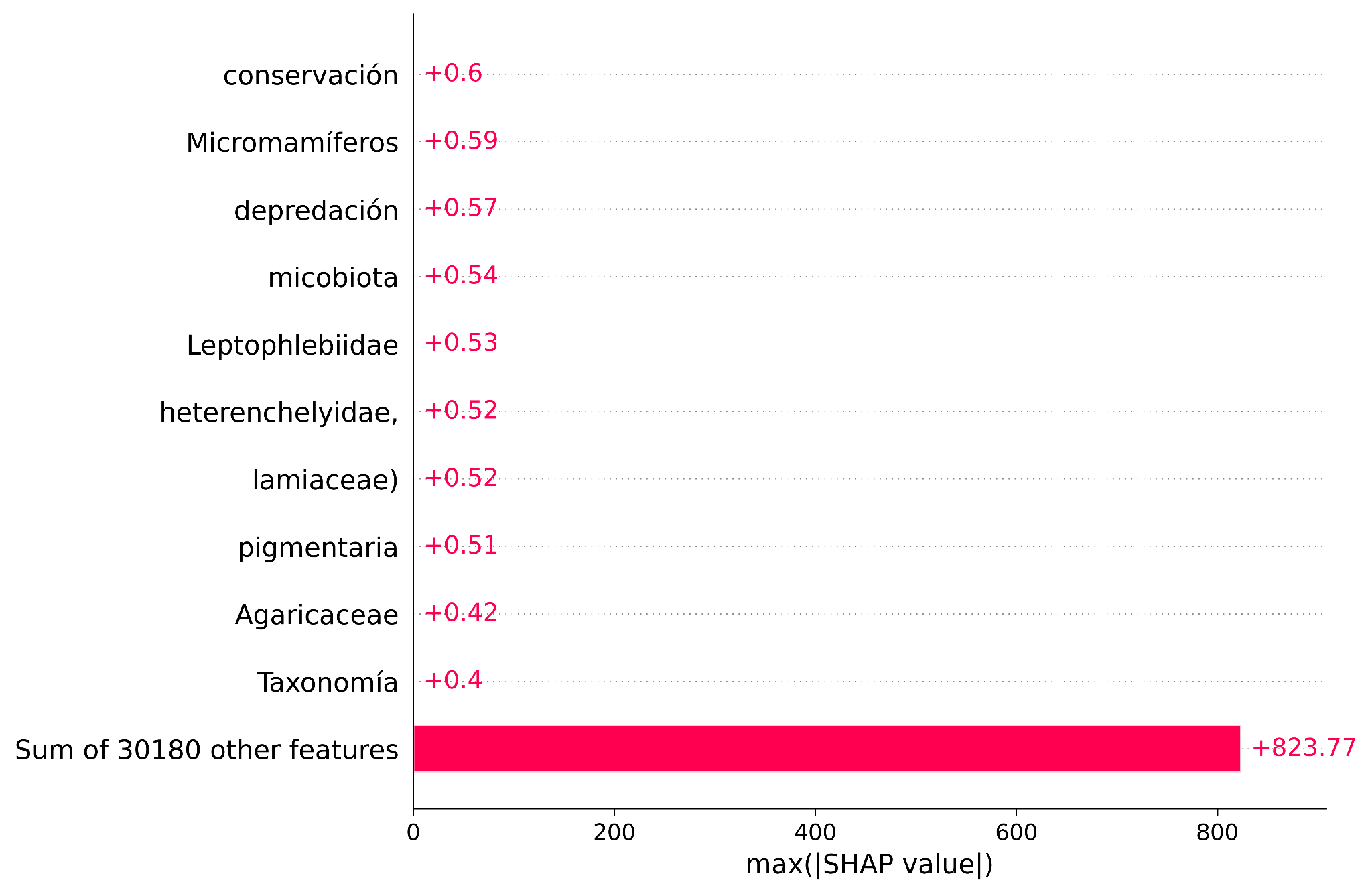

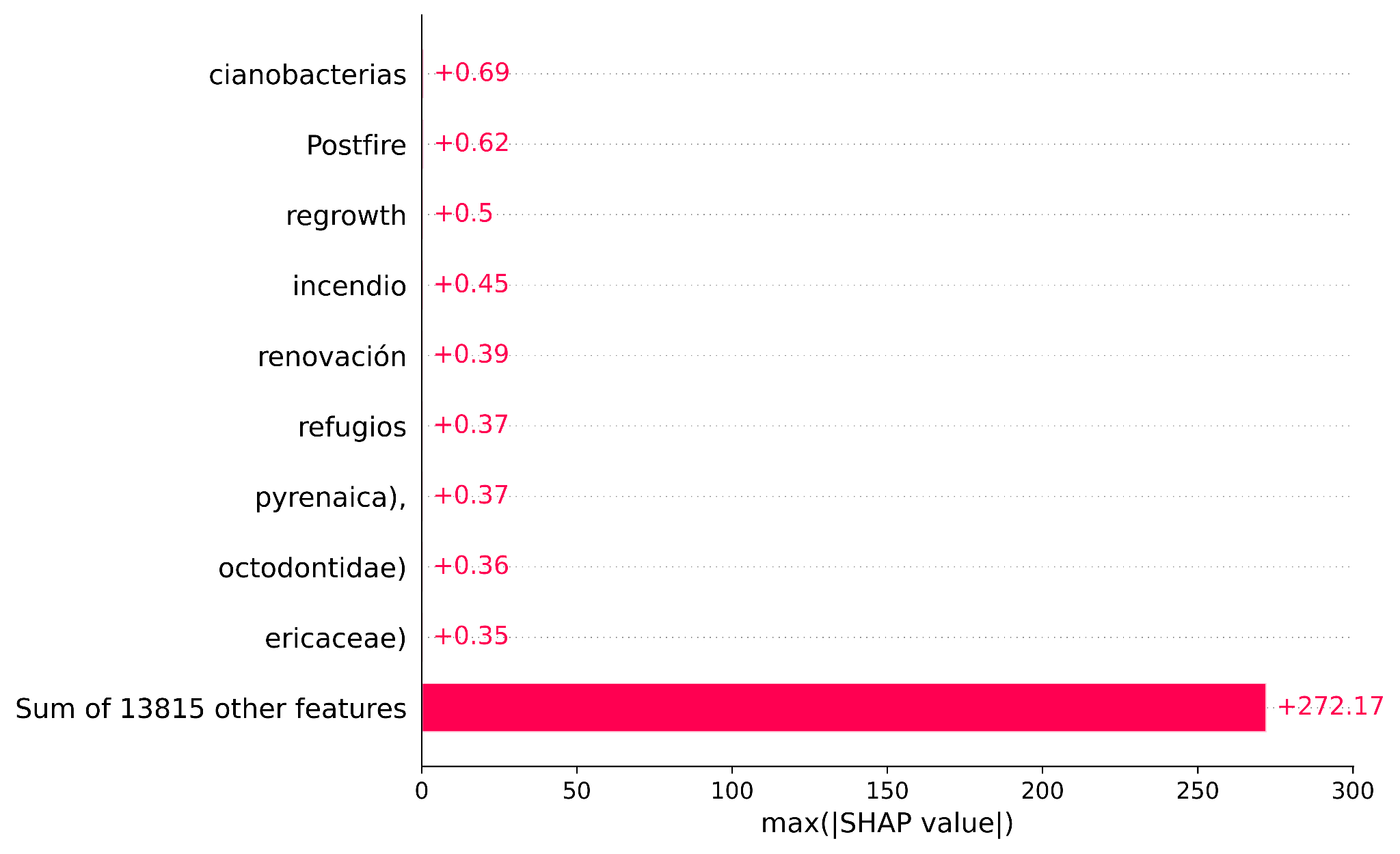

Supplement: Supplementary file 1 — Supplementary Material 1. [file 13750_2025_370_MOESM1_ESM.docx]
